# Effects of Different Telemonitoring Strategies on Chronic Heart Failure Care: Systematic Review and Subgroup Meta-Analysis

**DOI:** 10.2196/20032

**Published:** 2020-11-13

**Authors:** Hang Ding, Sheau Huey Chen, Iain Edwards, Rajiv Jayasena, James Doecke, Jamie Layland, Ian A Yang, Andrew Maiorana

**Affiliations:** 1 RECOVER Injury Research Centre Faculty of Health and Behavioural Sciences The University of Queensland Brisbane Australia; 2 The Australian e-Health Research Centre Commonwealth Scientific & Industrial Research Organisation Brisbane Australia; 3 Prince Charles Hospital - Northside Clinic Unit School Faculty of Medicine The University of Queensland Brisbane Australia; 4 School of Physiotherapy and Exercise Science Curtin University Perth Australia; 5 Department of Community Health Peninsula Health Melbourne Australia; 6 The Australian e-Health Research Centre Commonwealth Scientific & Industrial Research Organisation Melbourne Australia; 7 Department of Cardiology Peninsula Health Melbourne Australia; 8 Peninsula Clinical School Monash University Melbourne Australia; 9 Department of Thoracic Medicine The Prince Charles Hospital The University of Queensland Brisbane Australia; 10 Allied Health Department and Advanced Heart Failure and Cardiac Transplant Service Fiona Stanley Hospital Perth Australia

**Keywords:** telehealth, telemonitoring, mobile health, chronic heart failure, systematic review, meta-analysis

## Abstract

**Background:**

Telemonitoring studies in chronic heart failure are characterized by mixed mortality and hospitalization outcomes, which have deterred the uptake of telemonitoring in clinical practice. These mixed outcomes may reflect the diverse range of patient management strategies incorporated in telemonitoring. To address this, we compared the effects of different telemonitoring strategies on clinical outcomes.

**Objective:**

The aim of this systematic review and subgroup meta-analysis was to identify noninvasive telemonitoring strategies attributing to improvements in all-cause mortality or hospitalization outcomes for patients with chronic heart failure.

**Methods:**

We reviewed and analyzed telemonitoring strategies from randomized controlled trials (RCTs) comparing telemonitoring intervention with usual care. For each strategy, we examined whether RCTs that applied the strategy in the telemonitoring intervention (subgroup 1) resulted in a significantly lower risk ratio (RR) of all-cause mortality or incidence rate ratio (IRR) of all-cause hospitalization compared with RCTs that did not apply this strategy (subgroup 2).

**Results:**

We included 26 RCTs (N=11,450) incorporating 18 different telemonitoring strategies. RCTs that provided medication support were found to be associated with a significantly lower IRR value than RCTs that did not provide this type of support (*P*=.01; subgroup 1 IRR=0.83, 95% CI 0.72-0.95 vs subgroup 2 IRR=1.02, 95% CI 0.93-1.12). RCTs that applied mobile health were associated with a significantly lower IRR (*P*=.03; IRR=0.79, 95% CI 0.64-0.96 vs IRR=1.00, 95% CI 0.94-1.06) and RR (*P*=.01; RR=0.67, 95% CI 0.53-0.85 vs RR=0.95, 95% CI 0.84-1.07).

**Conclusions:**

Telemonitoring strategies involving medication support and mobile health were associated with improvements in all-cause mortality or hospitalization outcomes. These strategies should be prioritized in telemonitoring interventions for the management of patients with chronic heart failure.

## Introduction

Chronic heart failure (CHF) is a severe chronic disease [1] affecting over 26 million people worldwide [2]. Despite advances in modern medical therapy [3] and multidisciplinary clinical care [4], CHF continues to manifest a poor quality of life [5], frequent hospitalizations [6,7], low survival rates [8], and high health care expenditure [2]. Telemonitoring has been extensively studied as an innovative approach to enable care providers to remotely monitor patients at home and provide timely intervention in the event of clinical deterioration. Over the past two decades, many enabled care programs have been developed and evaluated, and several reviews have demonstrated the potential of using telemonitoring interventions to reduce mortality [9-11] and hospitalizations [9,11] in CHF care. However, the outcomes from individual randomized controlled trials (RCTs) are heterogeneous, with nonsignificant effects obtained in several large and well-designed RCTs [12-14]. Owing to these mixed outcomes, the use of telemonitoring in CHF care has been questioned [15,16] and has not yet been embraced in clinical recommendations [17,18].

Mixed outcomes in telemonitoring studies have been attributed to insufficient support from cardiologists, unsatisfactory patient compliance [19,20], low predictive power for clinical deterioration [14], and improvements in usual care [14,18]. However, these findings were limited to narrative analyses of individual telemonitoring studies. Several reviews have evaluated specific approaches to CHF care, including mobile health (mHealth) [21-23]; structured telephone [11,18], videophone, and interactive voice response devices [24]; education alone; pharmacist interventions; and clinical support by various care providers [25]. These reviews provide valuable insight into the effectiveness of specific types of interventions, but do not explain the mixed outcomes across telemonitoring interventions involving different components of care.

To address the existing knowledge gap, we conducted a systematic review and meta-analysis using a novel approach of evaluating the effect of different noninvasive telemonitoring strategies on reduced all-cause mortality and hospitalization to identify which strategies were associated with these outcomes.

## Methods

### Literature Search

This review was performed according to the Cochrane Collaboration methodological guidelines [26]. We conducted a literature search in the PubMed, EMBASE, CINAHL, and Cochrane Library databases, covering the publication period from January 1990 to February 2020. The publications were required to be (1) relevant to telehealth, telemedicine, telemonitoring, telecare, internet, mobile, smartphone, remote monitoring, or home monitoring; (2) involving patients with CHF; and (3) in the English language. An information specialist officer at the Commonwealth Scientific and Industrial Research Organization (Brisbane, Australia) and an expert librarian at Curtin University, Western Australia, Australia helped develop the bibliographies and conduct the database search (for a more complete description of our search strategy, see Multimedia Appendix 1).

Two investigators (HD and SC) independently reviewed the articles obtained. Disagreements between the two investigators were resolved by a third reviewer (AM or IE).

### Scope of Telemonitoring

In this review, we employed a hierarchical structure considering that telehealth encompasses telemonitoring, as well as eHealth care processes and communication, telemedicine, and mHealth [27]. We then defined the scope of telemonitoring as “the use of information technology to monitor patients at a distance,” as described by Meystre [28]. Finally, we included a telemonitoring intervention in the analysis if it involved “the transfer of physiological data such as blood pressure, weight, electrocardiographic signals, or oxygen saturation through technology such as telephone lines, broadband, satellite, or wireless networks” [27].

### Inclusion and Exclusion Criteria

This review focused on noninvasive telemonitoring interventions evaluated through an RCT. The inclusion criteria were: (1) studies evaluating telemonitoring for CHF for at least 3 months, (2) prospective RCTs comparing telemonitoring-based care with usual care, and (3) full peer-reviewed journal articles reporting outcomes of all-cause mortality or all-cause hospitalization. The exclusion criteria were: (1) articles reporting preliminary analysis outcomes; (2) studies with a sample size less than 50 (Multimedia Appendix 2), because, compared with large studies, small studies are often associated with a lower level of reporting quality [29], are more likely to be heterogeneous [30], and overestimate outcome effects [31]; and (3) telemonitoring via implantable devices, as these interventions often involve a different care paradigm to noninvasive devices and have been the subject of dedicated reviews [32,33].

### Telemonitoring Strategies Extracted

We extracted 18 telemonitoring strategies according to three categories: technology applications (6 strategies), care objectives (7 strategies), and care support methods (5 strategies) (Table 1).

**Table 1:** Extracted telemonitoring strategies for the subgroup meta-analysis on telemonitoring interventions for chronic heart failure (CHF).

Strategies		Descriptions
**Technology applications**		
	mHealth^a^ system (or combining with mHealth apps)	An mHealth system was used in the telemonitoring program, and the system involved a set of software apps mainly designed for mobile devices such as smartphones, personal digital assistants, and tablet computers.
	PC^b^-based system	A PC-based system was used in the telemonitoring program, which involved a set of software apps mainly designed for PCs.
	Weight scale	A device enabling participants to measure body weight and transfer the data to care providers in the telemonitoring program.
	Blood pressure monitor	A device enabling participants to measure blood pressure and transfer the data to care providers in the telemonitoring program.
	ECG^c^ monitoring device	A device enabling participants to record ECG and transfer the data to care providers in the telemonitoring program.
	Heart rate monitor	A device enabling participants to measure heart rate and transfer the data to care providers in the telemonitoring program.
**Care objectives**		
	Education	The telemonitoring program included a care objective/component involving CHF education. The education content could be provided via video clips, animation, or text messages.
	Daily weight monitoring	The telemonitoring program contained a care objective/component to assist the participants in daily weight monitoring. The assistance was delivered predominantly via automated messages and telephone calls.
	Diet	The telemonitoring program contained a care objective/component for improving dietary behavior recommended for CHF.
	Medication support	Clinical support was provided to optimally adjust medication therapy or support participants to adhere to the medication recommendations for CHF.
	Exercise	Exercise was monitored or assessed via electronic questionnaires in the program. Clinical interventions such as automated messages and telephone calls were provided to assist participants in conducting exercises according to clinical recommendations.
	Depression and anxiety	A care objective/component was specifically provided to address depression and anxiety in participants through the telemonitoring program.
	Monitoring symptoms	Participants used telemonitoring apps to record their CHF-related symptoms. Accordingly, care providers reviewed the recorded symptoms and provided interventions.
**Care support methods**		
	Collaborative care	Interventions and support for collaborative care were provided in the telemonitoring program, such as collaborative reviews, referrals, and communication for follow up.
	Physician support	Physicians were included in the telemonitoring program to provide clinical intervention to the participants.
	Nurse support	Nurses were included in the telemonitoring program to provide clinical intervention to the participants.
	Call center support	A call center was included in the telemonitoring program to provide support to the participants.
	Automated system	Automated systems were used to automatically monitor the participants’ data and provide reminders, alerts, and notifications to the participants.

^a^mHealth: mobile health.

^b^PC: personal computer.

^c^ECG: electrocardiogram.

### Review Outcomes

The risk ratio (RR) of all-cause mortality and the incidence rate ratio (IRR) of all-cause hospitalization in the RCTs were analyzed. The RR and IRR values in each RCT were calculated from the event counts of mortality and hospitalization. For each strategy, we divided the RCTs into two subgroups: RCTs that applied the strategy in the telemonitoring intervention (subgroup 1) and RCTs that did not apply the strategy (subgroup 2). We then compared the two subgroups (subgroup 1 vs subgroup 2), and examined whether the difference between the two groups in the RR and IRR outcomes was statistically significant.

### Meta-Analysis

In the meta-analysis, we used a random-effects model with the DerSimonian–Laird estimator [34,35], and report the RR, IRR, and 95% CI for each group. For RCTs with no events in one arm, we applied a continuity correction of 0.5. The heterogeneity of RCTs in each subgroup was examined by the *Q* test and I^2^ statistic [36,37]. The statistical significance of heterogeneity was determined by a relaxed *P* value of .10 (*P*_H_<.10) [38]. The I^2^ values of 25%, 50%, and 75% were used to reflect a low, moderate, and high level of heterogeneity, respectively [37]. To evaluate the risk of bias, a regression test was used to analyze the asymmetry of a funnel plot of the RR or IRR results in a subgroup [36]. The regression test was used to examine whether the outcomes of individual RCTs were related to the corresponding sampling variances [39]. A significant regression (*P*_F_<.05) indicated a significant risk of bias. The difference between the two groups (subgroup 1 vs subgroup 2) was evaluated by a Wald-type test [36], and statistical significance was determined if the corresponding two-sided *P* value was less than .05 (*P*_C_<.05). A mixed-effects model [36] was also used to evaluate the effects of potential confounders, including sex, age, or the severity measure of left ventricular ejection fraction (LVEF). The meta-analysis methods and tests were performed using RStudio Version 1.1.383 [40] associated with the “metafor” meta-analysis package (version 2.0) [36].

### Risk of Bias

A summary of the methodological risk of bias of the included studies was conducted in accordance with the Cochrane Handbook for Systematic Reviews of Interventions [26] by two investigators (HD, SC) using the risk of bias tool in the Cochrane Collaboration’s review-writing software RevMan 5.3. This involved reporting the following individual elements for the included RCTs: random sequence generation, allocation sequence concealment, blinding of participants and personnel, blinding of outcome assessment, completeness of outcome data, and selective outcome reporting. Each item was judged as being at a high, low, or unclear risk of bias. Studies were deemed to be at the highest risk of bias if they were scored at a high or unclear risk of bias for either the sequence generation or allocation concealment domains [26].

## Results

### Search Results

**Figure 1:**
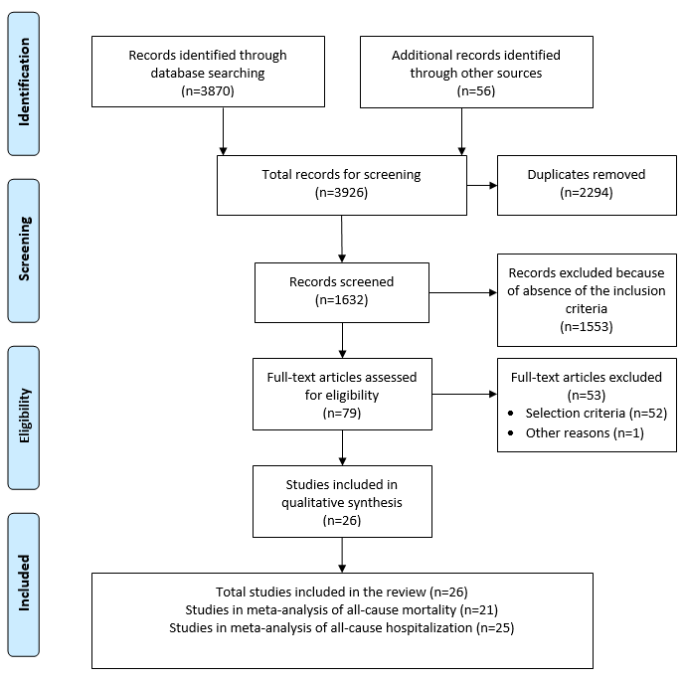
Flow diagram of study selection.

The literature search results are presented in Figure 1. We found 3870 records from the bibliographic search and 56 records from three existing systematic reviews [9,11,41] and a manual search, resulting in a total of 3926 records. After removing duplicates, we obtained 1632 articles for screening. In the screening process, we excluded 1553 articles because of absence of inclusion criteria and consequently obtained 79 articles for a full-text assessment. We then excluded 53 articles according to the inclusion and exclusion criteria, and one article because of its poor completion rate recognized by the authors [42]. Finally, this review included 26 RCTs. Among them, 25 RCTs provided all-cause hospitalization events and 21 RCTs provided mortality events.

**Figure 2:**
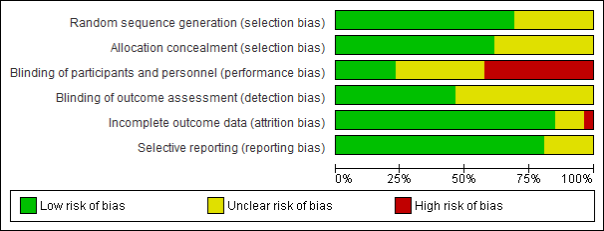
Risk of bias assessment. Authors' judgments about each methodological quality item are presented as percentages across all included studies.

**Figure 3:**
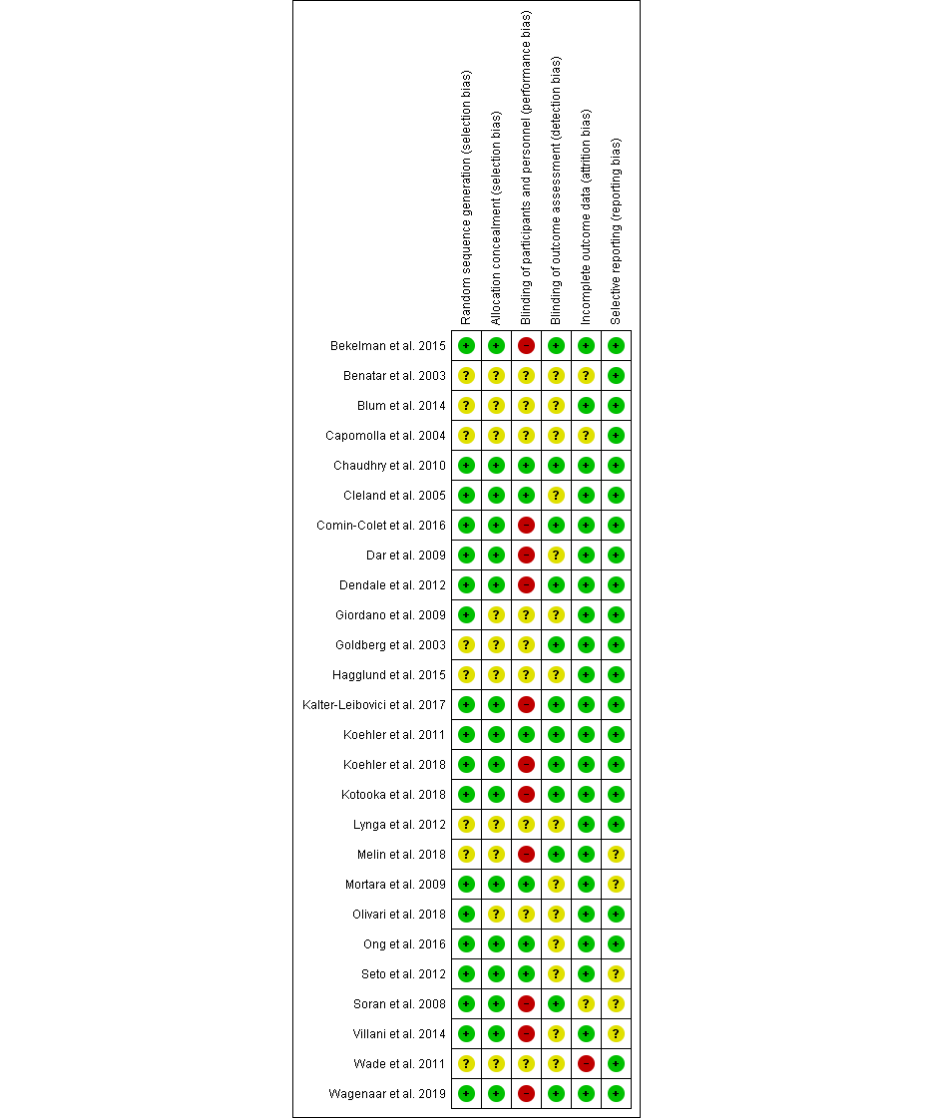
Risk of bias summary. Authors' judgements about each risk of bias item are summarized for each included study.

Among the assessment elements of bias risk, the blinding of participants and personnel was the least used method in the RCTs included (Figure 2). There were 11 RCTs that did not blind participants and personnel (Figure 3). Nine RCTs did not report their blinding status and only six RCTs used a blinding approach. The blinding of outcome assessment was the least reported element, and 14 RCTs (54%) had “unclear risk of bias.”

### Participant Characteristics

The 26 RCTs included 11,450 participants. The participants’ characteristics are shown in Table 2. The median age was 67.4 years and the median rate of male participants was 73.15% (8376/11,450). The participants generally had a significantly reduced (<40%) LVEF, with a median LVEF of 29.6%, and they experienced mild to moderate levels of symptoms, with a median New York Heart Association functional class score of 2.6. The median trial size of the RCTs was 290 participants and the median follow-up duration was 12 months.

**Table 2:** Participants’ characteristics in 26 randomized controlled trials included in the subgroup meta-analysis.

Characteristic	Median (IQR)
Age (years)	67.40 (65.08-72.75)
Trial size (N)	290 (180-675)
Follow-up duration (months)	12 (6-12)
Male (%)	73.15 (66.00-79.95)
LVEF^a^ (%)	29.60 (27.00-35.93)
NYHA^b^ class score	2.6 (2.3-2.8)

^a^LVEF: left ventricular ejection fraction.

^b^NYHA: New York Heart Association.

### Telemonitoring Strategies

We extracted 18 telemonitoring strategies from the 26 RCTs, as shown in Table 3. Some strategies were commonly used, such as telemonitoring weight scales (26/26, 100% RCTs), call-center support (24/26, 92%), and daily weight monitoring (25/26, 96%). Strategies that were not commonly used included nurse support (2/26, 8%), intervention for depression and anxiety (3/26, 12%), and exercise (3/26, 12%). The telemonitoring programs in the RCTs generally contained multiple strategies, with a mean of 8.7 strategies per care program.

**Table 3:** Telemonitoring strategies and randomized controlled trials included in the meta-analysis.

Reference	N	Care support method					Care objective									Technology application					
		Alerts	Nurse	Call Center	Physician	Collaborative		Ed^a^	Weight	Diet	Meds^b^	Ex^c^	DA^d^	Symptoms	PC^e^ app		mHealth^f^	Scale	BP^g^	HR^h^	ECG^i^
[43]	384	0	0	1	1	1		1	1	1	1	0	1	1	0		0	1	1	1	0
[44]	216	0	0	1	1	0		0	0	0	1	0	0	0	0		0	1	1	1	0
[45]	156	0	0	1	0	0		0	1	0	1	0	0	0	0		1	1	1	1	0
[46]	133	1	0	1	1	0		1	1	1	1	1	0	1	1		1	1	1	1	0
[12]	1653	0	0	1	1	0		1	1	0	0	0	0	1	0		0	1	0	0	0
[47]	248	0	0	1	1	0		0	1	0	0	0	0	0	0		0	1	1	1	1
[48]	178	1	0	1	1	1		0	1	0	1	0	0	1	0		1	1	1	1	0
[49]	182	0	0	1	1	0		0	1	0	1	0	0	1	0		0	1	1	0	0
[50]	160	0	1	1	1	1		0	1	1	0	1	0	0	0		1	1	1	1	0
[51]	460	0	1	1	1	0		1	1	1	1	0	0	1	0		1	1	1	0	1
[52]	280	0	0	1	1	0		0	1	0	0	0	0	1	0		1	1	0	0	0
[53]	72	1	0	1	1	0		1	1	0	1	0	0	1	0		1	1	0	0	0
[54]	1360	0	0	1	1	1		0	1	1	1	0	0	0	1		0	1	1	1	0
[55]	1538	0	0	1	1	1		1	1	0	1	0	0	1	0		1	1	1	1	1
[13]	710	0	0	1	1	0		0	1	0	0	0	0	1	0		1	1	1	0	1
[56]	181	0	0	1	1	0		0	1	0	0	0	0	0	0		0	1	1	1	0
[57]	319	1	0	1	0	0		0	1	0	1	0	0	0	0		0	1	0	0	0
[58]	72	1	0	1	1	0		1	1	1	1	0	0	1	0		1	1	0	0	0
[59]	261	0	0	1	1	0		1	1	0	0	0	0	1	0		1	1	1	1	1
[60]	339	0	0	1	1	0		0	1	0	0	0	0	0	0		0	1	1	1	1
[14]	1437	0	0	1	0	0		1	1	0	0	0	0	1	0		0	1	1	1	0
[61]	100	1	0	1	1	0		0	1	0	1	0	0	1	0		1	1	1	0	1
[62]	315	0	0	1	1	1		1	1	0	1	0	0	1	0		0	1	0	0	0
[63]	80	0	0	0	1	0		0	1	0	0	0	1	1	0		1	1	1	0	1
[64]	316	0	0	1	0	0		1	1	0	1	1	0	0	0		0	1	1	0	0
[65]	300	1	1	0	0	1		1	1	0	1	0	0	1	0		0	1	1	1	0
Total	11,450	7	3	24	21	7		12	25	6	16	3	2	17	2		13	26	20	14	8

^a^Ed: education.

^b^Meds: medication.

^c^Ex: exercise.

^d^D/A: depression and anxiety.

^e^PC: personal computer.

^f^mHealth: mobile health.

^g^BP: blood pressure.

^h^HR: heart rate.

^i^ECG: electrocardiogram.

### Overall Effectiveness of Telemonitoring

**Figure 4:**
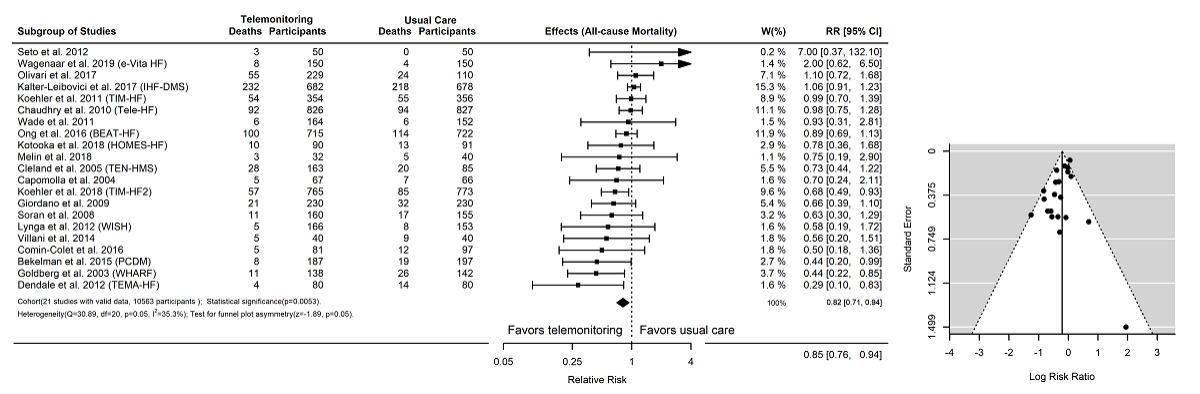
Event counts and effectiveness of telemonitoring interventions on all-cause mortality. There were 20 randomized controlled trials (N=10,263) with mortality event counts in the subgroup meta-analysis. RR: relative risk.

**Figure 5:**
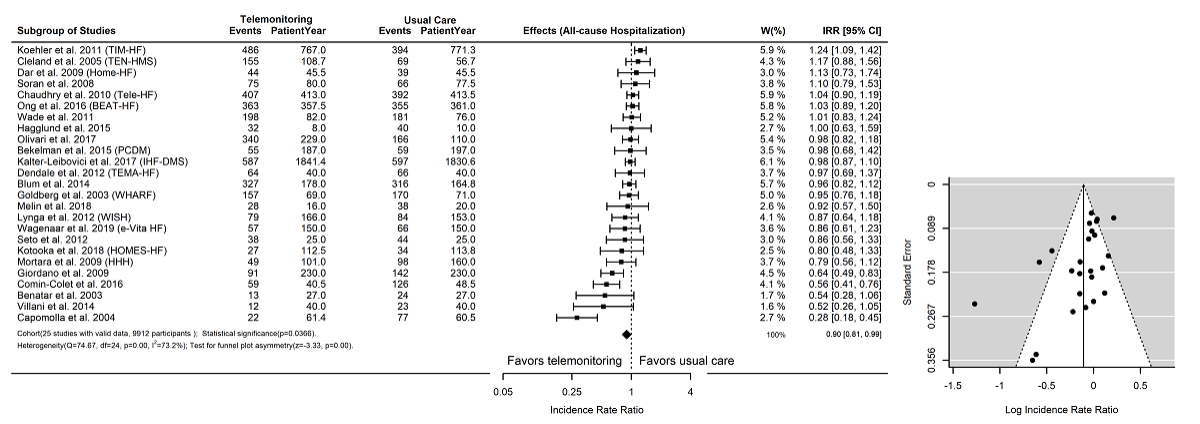
Event counts and effectiveness of telemonitoring interventions on all-cause hospitalization. There were 24 randomized controlled trials (N=9612) with hospitalization event counts in the subgroup meta-analysis. IRR: incidence rate ratio.

There were 21 RCTs (n=10,536) with event counts of all-cause mortality and 25 RCTs (n=9912) with event counts of all-cause hospitalization. The outcomes of mortality (RR) and hospitalization (IRR) with 95% CIs are shown in Figure 4 and Figure 5, respectively. Overall, telemonitoring interventions were found to be more effective than usual care on reducing all-cause mortality (RR=0.85, 95% CI 0.76-0.94, *P*=.01) and all-cause hospitalizations (IRR=0.90, 95% CI 0.81-0.99, *P*=.04). The outcomes of both RR and IRR were heterogeneous (*P*_H_=.001), with a low-to-moderate level of heterogeneity (I^2^=35.3%) in the RR outcomes and a moderate-to-high level of heterogeneity (I^2^=73.2%) in the IRR outcomes. In the funnel plot–based test, the risk of bias was significant for both RR (z=1.89, *P*_F_=.001 and IRR (z=3.33, *P*_F_=.001) outcomes. We also used the mixed-effects model to adjust for sex, age, or LVEF, but did not find significant differences in these results.

### Subgroup Comparison of Telemonitoring Strategies

The subgroup of RCTs that provided medication support (subgroup 1, 15 RCTs, n=4563, IRR=0.83, 95% CI 0.72-0.95) was found to be associated with a significantly (*P*=.01) lower IRR of all-cause hospitalization than the comparison subgroup of RCTs that did not apply this strategy (subgroup 2, 10 RCTs, n=5349, IRR=1.02, 95% CI 0.93-1.12), as shown in Tables 4-6 and Multimedia Appendix 3. Within the subgroup of RCTs that provided medication support, the telemonitoring interventions were found to be more effective than usual care on reducing hospitalizations (15 RCTs, n=4563, IRR=0.83, 95% CI 0.72-0.95, *P*=.01). The IRR outcomes in both subgroups were heterogeneous (Multimedia Appendix 3 and Multimedia Appendix 4). The outcomes in the comparison subgroup of RCTs that did not apply this strategy were associated with the risk of bias.

Similarly, the subgroup of mHealth (subgroup 1, 12 RCTs, n=2662, IRR=0.79, 95% CI 0.64-0.96) was associated with a significantly (*P*=.03) lower IRR of all-cause hospitalization than the comparison subgroup (subgroup 2, 13 RCTs, n=7250, IRR=1.00, 95% CI 0.94-1.06), as shown in Tables 4-6 and Multimedia Appendix 5. Within the mHealth subgroup, the telemonitoring interventions were found to be significantly more effective than usual care on reducing all-cause hospitalizations (subgroup 1, 12 RCTs, n=2662, IRR=0.79, 95% CI 0.64-0.96, *P*=.03). The IRR outcomes in the mHealth subgroup were heterogeneous and were associated with a risk of bias.

**Table 4:** Subgroup meta-analysis to examine the effect of telemonitoring strategies on all-cause hospitalization for randomized controlled trials (RCTs) that applied the strategy in the telemonitoring intervention (subgroup 1).

Strategies		RCTs (N participants)	Effect				Heterogeneity					Funnel test *Z* (*P* value)
			IRR^a^ (95% CI)		*P* value	*Q* (*P* value)		I^2^				
**Technology application**												
	PC^b^-based system	2 (1493)	0.54 (0.16-1.81)		.32	25.07 (<.001)		96.0%		0.00 (<.001)		
	Blood Pressure Monitor	19 (7201)	0.87 (0.77-0.98)		.02	72.50 (<.001)		75.2%		–3.30 (.001)		
	ECG^c^ Monitor	7 (2198)	0.91 (0.73-1.12)		.37	27.56 (<.001)		78.2%		–2.01 (.04)		
	Telemonitoring Weight Scale	25 (9912)	0.90 (0.83-0.99)		.03	74.67 (<.001)		67.9%		–3.24 (.001)		
	Heart Rate Monitor	13 (5353)	0.85 (0.74-0.97)		.02	44.39 (<.001)		73.0%		–2.92 (.003)		
	Mobile Health System	12 (2662)	0.79 (0.64-0.96)		.02	64.40 (<.001)		82.9%		–2.16 (.03)		
**Care objective**												
	Education	10 (5103)	0.86 (0.72-1.02)		.10	39.11 (<.001)		77.0%		–1.82 (.07)		
	Daily Weight Monitoring	24 (9696)	0.91 (0.83-1.00)		.05	71.77 (<.001)		68.0%		–2.90 (.004)		
	Monitoring Symptoms	16 (6617)	0.86 (0.74-0.99)	.04			68.81 (<.001)		78.2%		–2.38 (.02)	
	Medication	15 (4563)	0.83 (0.72-0.95)	.01			47.71 (<.001)		70.7%		–1.55 (.12)	
	Diet	6 (2569)	0.75 (0.56-1.02)	.07			31.76 (<.001)		84.3%		–1.07 (.29)	
	Exercise	3 (609)	0.67 (0.35-1.29)	.24			24.38 (<.001)		91.8%		–1.70 (.09)	
	Depression and Anxiety	2 (464)	0.77 (0.42-1.40)	.39			2.47 (.11)		59.5%		0.00 (<.001)	
**Care support method**												
	Call Center Support	23 (9532)	0.91 (0.83-1.00)	.06			71.15 (<.001)		69.1%		–2.83 (.005)	
	Physician Support	20 (7384)	0.88 (0.78-0.99)	.03			72.83 (<.001)		73.9%		–2.72 (.01)	
	Automated Alerts	7 (1174)	0.72 (0.53-0.96)	.03			23.59 (<.001)		74.6%		–0.25 (.80)	
	Collaborative Care Support	6 (2697)	0.89 (0.75-1.07)	.22			12.32 (.03)		59.4%		–0.21 (.83)	
	Nurse Support	3 (920)	0.80 (0.61-1.03)	.08			3.99 (.13)		49.9%		1.90 (.06)	

^a^IRR: incidence rate ratio.

^b^PC: personal computer.

^c^ECG: electrocardiogram.

**Table 5:** Subgroup meta-analysis to examine the effect of telemonitoring strategies on all-cause hospitalization for randomized controlled trials (RCTs) that did not apply the strategy in the telemonitoring intervention (subgroup 2).

Strategies		RCTs (N participants)	Effect			Heterogeneity				Funnel test Z (*P* value)	
			IRR^a^ (95% CI)		*P* value		*Q* (*P* value)		I^2^		
**Technology application**											
	PC^b^-based System	23 (8419)	I0.94 (0.86- 1.01)	.13		48.12 (<.001)		54.3%			–2.68 (.007)
	Blood Pressure Monitor	6 (2711)	1.00 (0.91- 1.10)	.99		1.78 (.87)		0.0%			–0.53 (.59)
	ECG^c^ Monitor	18 (7714)	0.90 (0.81- 0.99)	.05		45.19 (<.001)		62.4%			–2.46 (.01)
	Heart Rate Monitor	12 (4559)	0.96 (0.85-1.08)	.55		26.57 (<.001)		58.6%			–1.70 (.09)
	Mobile Health System	13 (7250)	1.00 (0.94-1.06)	.99		7.90 (.79)		0.0%			–1.04 (.30)
**Care objective**											
	Education	15 (4809)	0.93 (0.84- 1.03)	.21		34.76 (<.001)		59.7%			–2.53 (.01)
	Daily Weight Monitoring	1 (216)	N/A^d^	N/A		N/A		N/A			N/A
	Monitoring Symptoms	9 (3295)	0.97 (0.91- 1.04)	.44		5.85 (.66)		0.0%			–1.13 (.26)
	Medication	10 (5349)	1.02 (0.93-1.12)	.67		15.72 (.07)		42.7%			–2.79 (.01)
	Diet	19 (7343)	0.96 (0.88-1.04)	.35		37.33 (<.001)		51.8%			–2.98 (.003)
	Exercise	22 (9303)	0.94 (0.86-1.01)	.12		48.04 (<.001)		56.3%			–2.58 (.01)
	Depression and Anxiety	23 (9448)	0.91 (0.83-0.99)	.05		71.60 (<.001)		69.3%			–2.94 (.003)
**Care support method**											
	Call Center Support	2 (380)	0.74 (0.47-1.16)	.19		1.59 (.20)		37.2%			0.00 (<.001)
	Physician Support	5 (2528)	0.98 (0.90-1.06)	.65		1.78 (.77)		0.0%			–1.05 (.29)
	Automated Alerts	18 (8738)	0.98 (0.91-1.05)	.61		33.10 (.01)		48.6%			–2.39 (.02)
	Collaborative Care Support	19 (7215)	0.90 (0.81-1.01)	.08		61.24 (<.001)		70.6%			–3.35 (.001)
	Nurse Support	22 (8992)	0.92 (0.84- 1.01)	.10		64.19 (<.001)		67.3%			–3.32 (.001)

^a^IRR: risk ratio of mortality.

^b^PC: personal computer.

^c^ECG: electrocardiogram.

^d^N/A: not applicable due to insufficient data.

**Table 6:** Comparison of the effect of telemonitoring strategies on all-cause hospitalization and all-cause mortality between subgroup 1 and subgroup 2.

Strategies		All-cause hospitalization *P* value		All-cause mortality *P* value	
**Technology application**					
	Blood Pressure Monitor		.08		.46
	ECG^a^ Monitor		.98		.89
	Heart Rate Monitor		.19		.92
	Mobile Health System		.03		.01
**Care objectives**					
	Education		.45		.92
	Monitoring Symptoms		.13		.40
	Medication		.02		.59
	Diet		.13		.33
	Exercise		.33		.28
	Depression and Anxiety		N/A^b^		.09
**Care support method**					
	Call Center Support		.37		.73
	Physician Support		.14		.35
	Automated Alerts		.05		.99
	Collaborative Care Support		.92		.28
	Nurse Support		.29		.66

^a^ECG: electrocardiogram.

^b^N/A: not applicable due to insufficient data for comparison.

In analysis of all-cause mortality, the mHealth subgroup (subgroup 1, 10 RCTs, n=3711, RR=0.67, 95% CI 0.53-0.85) was also associated with a significantly (*P*=.01) lower RR than the comparison subgroup (subgroup 2, 11 RCTs, n=6852, RR=0.95, 95% CI 0.84-1.07), as shown in Tables 6-8 and Multimedia Appendix 6. Within the mHealth subgroup, the telemonitoring interventions were significantly more effective than usual care on reducing all-cause mortality (subgroup 1, 10 RCTs, n=3711, RR=0.67, 95% CI 0.53-0.85, *P*<.001). No significant heterogeneity was detected in both the mHealth subgroup and comparison subgroup. A significant risk of bias (*P*_F_=.01) was found in the comparison subgroup.

In the subgroup comparison of RR and IRR outcomes, we also used the mixed-effects model to adjust for sex, age, or LVEF, but did not find significant improvements in these RR and IRR analysis results.

**Table 7:** Subgroup meta-analysis to examine the effect of telemonitoring strategies on mortality in randomized controlled trials (RCTs) that applied the strategy in the telemonitoring intervention (subgroup 1).

Strategies		RCTs (N participants)	Effect		Heterogeneity (*P* value) and Funnel Test (*P* value)				Funnel test Z (*P* value)
			RR^a^ (95% CI)	*P* value	*Q* (*P* value)	I^2^			
**Technology application**									
	PC^b^-based System	2 (1493)	1.05 (0.90-1.21)	.52	0.52 (.47)	0.0%		0.00 (<.001)	
	Blood Pressure Monitor	16 (7924)	0.83 (0.71-0.98)	.03	24.22 (.06)		38.1%		–1.03 (.30)
	ECG^c^ Monitor	7 (3475)	0.82 (0.66-1.01)	.07	7.88 (.24)		23.8%		0.66 (.51)
	Telemonitoring Weight Scale	21 (10563)	0.82 (0.71-0.94)	.005	30.89 (.05)		35.3%		–1.89 (.06)
	Heart Rate Monitor	11 (6258)	0.82 (0.67-1.00)	.05	19.66 (.03)		49.1%		–1.69 (.09)
	Mobile Health System	10 (3711)	0.67 (0.53-0.85)	.001	11.58 (.23)		22.3%		–0.27 (.78)
**Care objectives**									
	Education	9 (6308)	0.81 (0.70-0.93)	.004	7.00 (.53)		0.0%		–1.37 (.17)
	Daily Weight Monitoring	21 (10563)	0.82 (0.71-0.94)	.005	30.89 (.05)		35.3%		–1.89 (.06)
	Monitoring Symptoms	14 (7640)	0.78 (0.66-0.92)	.004	17.33 (.18)		25.0%		–0.48 (.63)
	Medication	12 (5475)	0.77(0.60-0.98)	.04	18.60 (.06)		40.9%		0.12 (.90)
	Diet	6 (2569)	0.67 (0.43-1.03)	.07	12.60 (.02)		60.3%		–3.05 (.002)
	Exercise	3 (609)	0.56 (0.28-1.13)	.11	2.49 (.28)		19.5%		1.57 (.12)
	Depression and Anxiety	2 (464)	0.48 (0.26-0.90)	.02	0.12 (.73)		0.0%		0.00 (<.001)
**Care support method**									
	Call Center Support	19 (10183)	0.81 (0.70-0.93)	.005	28.23 (.05)		36.2%		–2.52 (.01)
	Physician Support	17 (8191)	0.78 (0.66-0.92)	.005	28.47 (.02)		43.8%		–2.43 (.01)
	Automated Alerts	6 (1102)	0.82 (0.48-1.39)	.46	5.68 (.33)		11.9%		1.67 (.09)
	Collaborative Care Support	7 (4235)	0.70 (0.48-1.01)	.06	18.75 (<.001)		68.0%		–1.14 (.26)
	Nurse Support	3 (920)	0.69 (0.29-1.67)	.42	5.76 (.05)		65.3%		0.23 (.81)

^a^RR: risk ratio.

^b^PC: personal computer.

^c^ECG: electrocardiogram.

**Table 8:** Subgroup meta-analysis to examine the effect of telemonitoring strategies on mortality in randomized controlled trials (RCTs) that did not apply the strategy in the telemonitoring intervention (subgroup 2).

Strategies		RCTs (N participants)	Effect		Heterogeneity		Funnel test Z (*P* value)
			RR^a^ (95% CI)	*P* value	Q (*P* value)	I^2^	
**Technology application**							
	PC^b^-based System	19 (9070)	0.79 (0.68-0.91)	.002	23.55 (.17)	23.6%	–1.08 (.28)
	Blood Pressure Monitor	5 (2639)	0.71 0.49-1.03)	.08	6.08 (.19)	34.3%	–1.80 (.07)
	ECG^c^ Monitor	14 (7088)	0.80 (0.66-0.97)	.03	21.59 (.06)	39.8%	–2.91 (.004)
	Heart Rate Monitor	10 (4305)	0.81 (0.65-0.99)	.04	10.50 (.31)	14.3%	–0.59 (.55)
	Mobile Health System	11 (6852)	0.95 (0.84-1.07)	.40	11.01 (.35)	9.2%	–1.67 (.09)
**Care objectives**							
	Education	12 (4255)	0.82 (0.65-1.04)	.11	20.66 (.03)	46.8%	–0.89 (.37)
	Monitoring Symptoms	7 (2923)	0.89 (0.69-1.13)	.35	8.88 (.18)	32.4%	–2.26 (.02)
	Medication	9 (5088)	0.84 (0.70-1.01)	.07	12.11 (.14)	33.9%	–2.58 (.01)
	Diet	15 (7994)	0.84 (0.73-0.96)	.02	16.50 (.28)	15.2%	–0.34 (.73)
	Exercise	18 (9954)	0.83 (0.72-0.96)	.01	26.24 (.07)	35.2%	–1.46 (.14)
	Depression and Anxiety	19 (10099)	0.84 (0.73-0.96)	.02	26.97 (.07)	33.3%	–1.42 (.17)
**Care support method**							
	Call Center Support	2 (380)	1.01 (0.29-3.54)	.98	2.63 (.10)	62.0%	0.00 (<.001)
	Physician Support	4 (2372)	0.90 (0.71-1.13)	.37	2.42 (.49)	0.0%	0.34 (.74)
	Automated Alerts	15 (9461)	0.81 (0.70-0.94)	.008	25.03 (.03)	44.1%	–3.76 (<.001)
	Collaborative Care Support	14 (6328)	0.87 (0.77-0.99)	.04	11.76 (.54)	0.0%	–1.01 (.31)
	Nurse Support	18 (9643)	0.85 (0.74-0.96)	.02	23.23 (.14)	26.8%	–2.36 (.02)

^a^RR: relative risk.

^b^PC: personal computer.

^c^ECG: electrocardiogram.

## Discussion

### Principal Findings

In this systematic review and meta-analysis, we evaluated 18 telemonitoring strategies in 26 RCTs. In addition to a traditional meta-analysis for overall effectiveness, we used a subgroup comparison method to analyze the effects of different telemonitoring components on clinical outcomes. We found that the telemonitoring strategy of providing medication support was associated with reduced all-cause hospitalization, whereas mHealth systems were associated with both reduced all-cause hospitalization and reduced all-cause mortality. Therefore, our review provides unique insight into specific telemonitoring strategies associated with improved clinical outcomes, which will help inform future telemonitoring interventions.

The positive findings related to the medication support strategy underscore the importance of medication therapy in telemonitoring interventions for CHF care. Strong evidence supports the role of modern pharmacological therapy in CHF management for delaying CHF deterioration [66,67], and reducing mortality and hospitalizations [18,67]. However, the therapeutic benefits are often limited by suboptimal patient adherence [68] and this limitation is not addressed by traditional face-to-face consultations [25]. Our findings suggest that the use of telemonitoring improves the efficacy of medication therapy, possibly through frequent reinforcement of compliance, leading to reduced episodes of clinical deterioration requiring hospitalization. Further research on optimizing medication therapy and underlying care processes in telemonitoring interventions is warranted to improve clinical outcomes in CHF care.

Using the subgroup comparison method, we also found that the strategy of providing telemonitoring interventions through an mHealth system was associated with a significant improvement in both all-cause mortality and hospitalization (or corresponding RR and IRR) outcomes. These positive findings could be supported by several unique advantages of using mHealth for general chronic disease care, including ease of use, portability, and real-time communication [69-71]. These advantages have been shown to improve the underlying care processes of patients’ self-management [72], care engagement [73,74], and medication adherence in CHF [75]. Therefore, our positive findings support delivering telemonitoring interventions through mHealth platforms, consistent with the increasing trend in using smartphones and computer tablets for the primary and secondary prevention of chronic disease [76,77].

Three recent reviews of mHealth in CHF management have resulted in inconsistent outcomes and, consequently, were unable to conclude significant clinical benefits [21,23,78]. In contrast to these traditional reviews, each intervention program in our mHealth subgroup combined both telemonitoring and mHealth interventions. Our positive finding indicates that simple mHealth apps without telemonitoring (enabling care providers to provide timely clinical intervention), such as apps only focusing on self-management or education, were insufficient to improve clinical outcomes. Similarly, this finding suggests that telemonitoring programs focusing on clinical assessment and intervention, but not delivered through an mHealth environment, fail to engage patients with CHF in self-management to the same extent as those provided via mHealth. Therefore, our finding warrants future research on comprehensive care programs combining telemonitoring and mHealth to improve both timely clinical intervention and patient engagement in CHF care.

As a part of our evaluation, we also conducted a traditional meta-analysis to evaluate the overall effectiveness of all of the telemonitoring interventions in the RCTs included in this review. We found that telemonitoring interventions were more effective than usual care on reducing both all-cause mortality and all-cause hospitalizations. This finding adds evidence to support telemonitoring interventions for CHF care generally. In our review, invasive telemonitoring interventions and small RCTs were excluded. These exclusions may have refined the selection of telemonitoring studies, leading to the significant findings, in contrast to the three previous mHealth reviews with inconclusive findings [21,23,78].

It is also important to note that several strategies such as daily weight monitoring, call center support, and exercise contained limited numbers of RCTs in the subgroup or comparison group. The evaluation of these strategies was therefore limited by our subgroup comparison method. However, these strategies should not be overlooked, and further research on their contributions to CHF care, such as improving patient adherence to daily weight monitoring and level of exercise, remains essential to continuously improve telemonitoring outcomes in future studies.

### Limitations

Because the objective of our review was to evaluate different telemonitoring strategies, our meta-analysis did not rigorously exclude RCTs with risk of bias, although we did exclude studies with small sample sizes. In addition, this review was an exploratory study, and hence we did not adjust the *P* value in the multiple comparisons of the telemonitoring strategies.

### Conclusions

The issues of mixed mortality and hospitalization outcomes have deterred the adoption of telemonitoring in CHF care. To address this issue, this review extensively investigated strategy-related factors associated with improvements in the outcomes, and found that the strategies of (1) providing medication support and (2) combining telemonitoring interventions through mHealth were associated with a significant improvement in all-cause mortality or hospitalizations. Importantly, these findings emphasize the importance of prioritizing medication therapy and patient engagement through mHealth apps in future telemonitoring interventions for CHF care.

## Appendix

Multimedia Appendix 1 Databases and search strategy in the literature search.

Multimedia Appendix 2 The list of excluded studies with the reason for exclusion.

Multimedia Appendix 3 Effectiveness of the strategy of providing medication support on reducing the risk of all-cause hospitalization. The subgroup of randomized controlled trials (RCTs) that provided medication support was compared with the subgroup of RCTs that did not provide medication support.

Multimedia Appendix 4 Effectiveness of the strategy of providing medication support on reducing the risk of all-cause mortality. The subgroup of randomized controlled trials (RCTs) that provided medication support were compared with the subgroup of RCTs that did not provide medication support.

Multimedia Appendix 5 Effectiveness of the strategy of combining with mobile health (mHealth), or applying an mHealth system, on reducing the risk of all-cause hospitalization. The subgroup of randomized controlled trials (RCTs) that applied the mHealth strategy were compared with the subgroup of RCTs that did not apply the strategy.

Multimedia Appendix 6 Effectiveness of the strategy of combining with mobile health (mHealth), or applying an mHealth system, on reducing the risk of all-cause mortality. The subgroup of randomized controlled trials (RCTs) that applied the mHealth strategy were compared with the subgroup of RCTs that did not apply the strategy.

## Abbreviations

CHF: chronic heart failure
IRR: incidence rate ratio
LVEF: left ventricular ejection fraction
mHealth: mobile health
RCT: randomized controlled trial
RR: risk ratio

## References

[1] Ponikowski P, Anker SD, AlHabib KF, Cowie MR, Force TL, Hu S, Jaarsma T, Krum H, Rastogi V, Rohde LE, Samal UC, Shimokawa H, Budi Siswanto B, Sliwa K, Filippatos G (2014). Heart failure: preventing disease and death worldwide. ESC Heart Fail.

[2] Savarese G, Lund LH (2017). Global Public Health Burden of Heart Failure. Card Fail Rev.

[3] Burnett H, Earley A, Voors AA, Senni M, McMurray JJ, Deschaseaux C, Cope S (2017). Thirty Years of Evidence on the Efficacy of Drug Treatments for Chronic Heart Failure With Reduced Ejection Fraction: A Network Meta-Analysis. Circ Heart Fail.

[4] Riley JP, Masters J (2016). Practical multidisciplinary approaches to heart failure management for improved patient outcome. Eur Heart J Suppl.

[5] Jeon Y, Kraus SG, Jowsey T, Glasgow NJ (2010). The experience of living with chronic heart failure: a narrative review of qualitative studies. BMC Health Serv Res.

[6] Desai AS, Stevenson LW (2012). Rehospitalization for Heart Failure. Circulation.

[7] Butler J, Kalogeropoulos A (2008). Worsening heart failure hospitalization epidemic we do not know how to prevent and we do not know how to treat!. J Am Coll Cardiol.

[8] Taylor CJ, Ryan R, Nichols L, Gale N, Hobbs FR, Marshall T (2017). Survival following a diagnosis of heart failure in primary care. Fam Pract.

[9] Klersy C, De Silvestri A, Gabutti G, Regoli F, Auricchio A (2009). A meta-analysis of remote monitoring of heart failure patients. J Am Coll Cardiol.

[10] Kitsiou S, Paré G, Jaana M (2015). Effects of home telemonitoring interventions on patients with chronic heart failure: an overview of systematic reviews. J Med Internet Res.

[11] Clark RA, Inglis SC, McAlister FA, Cleland JGF, Stewart S (2007). Telemonitoring or structured telephone support programmes for patients with chronic heart failure: systematic review and meta-analysis. BMJ.

[12] Chaudhry SI, Mattera JA, Curtis JP, Spertus JA, Herrin J, Lin Z, Phillips CO, Hodshon BV, Cooper LS, Krumholz HM (2010). Telemonitoring in patients with heart failure. N Engl J Med.

[13] Koehler F, Winkler S, Schieber M, Sechtem U, Stangl K, Böhm M, Boll H, Baumann G, Honold M, Koehler K, Gelbrich G, Kirwan B, Anker SD, Telemedical Interventional Monitoring in Heart Failure Investigators (2011). Impact of remote telemedical management on mortality and hospitalizations in ambulatory patients with chronic heart failure: the telemedical interventional monitoring in heart failure study. Circulation.

[14] Ong MK, Romano PS, Edgington S, Aronow HU, Auerbach AD, Black JT, De Marco T, Escarce JJ, Evangelista LS, Hanna B, Ganiats TG, Greenberg BH, Greenfield S, Kaplan SH, Kimchi A, Liu H, Lombardo D, Mangione CM, Sadeghi B, Sadeghi B, Sarrafzadeh M, Tong K, Fonarow GC, Better Effectiveness After Transition–Heart Failure (BEAT-HF) Research Group (2016). Effectiveness of Remote Patient Monitoring After Discharge of Hospitalized Patients With Heart Failure: The Better Effectiveness After Transition -- Heart Failure (BEAT-HF) Randomized Clinical Trial. JAMA Intern Med.

[15] Bui AL, Horwich TB, Fonarow GC (2011). Epidemiology and risk profile of heart failure. Nat Rev Cardiol.

[16] Bui AL, Fonarow GC (2012). Home monitoring for heart failure management. J Am Coll Cardiol.

[17] Ponikowski P, Voors AA, Anker SD, Bueno H, Cleland J, Coats A, Falk V, González-Juanatey JR, Harjola V, Jankowska E, Jessup M, Linde C, Nihoyannopoulos P, Parissis JT, Pieske B, Riley JP, Rosano G, Ruilope LM, Ruschitzka F, Rutten F, van der Meer P, ESC Scientific Document Group (2016). 2016 ESC Guidelines for the diagnosis and treatment of acute and chronic heart failure: The Task Force for the diagnosis and treatment of acute and chronic heart failure of the European Society of Cardiology (ESC)Developed with the special contribution of the Heart Failure Association (HFA) of the ESC. Eur Heart J.

[18] Inglis SC, Clark RA, Dierckx R, Prieto-Merino D, Cleland JGF (2017). Structured telephone support or non-invasive telemonitoring for patients with heart failure. Heart.

[19] Chaudhry S, Mattera J, Curtis J, Spertus J, Herrin J, Lin Z (2010). Telemonitoring to improve outcomes after heart failure hospitalization: a randomized controlled trial. Circulation; 2010 Clinical Trial/Clinical Science Abstracts.

[20] Ding H, Jayasena R, Maiorana A, Dowling A, Chen SH, Karunanithi M, Layland J, Edwards I (2017). Innovative Telemonitoring Enhanced Care Programme for Chronic Heart Failure (ITEC-CHF) to improve guideline compliance and collaborative care: protocol of a multicentre randomised controlled trial. BMJ Open.

[21] Cajita MI, Gleason KT, Han H (2016). A Systematic Review of mHealth-Based Heart Failure Interventions. J Cardiovasc Nurs.

[22] Masterson Creber RM, Maurer MS, Reading M, Hiraldo G, Hickey KT, Iribarren S (2016). Review and Analysis of Existing Mobile Phone Apps to Support Heart Failure Symptom Monitoring and Self-Care Management Using the Mobile Application Rating Scale (MARS). JMIR Mhealth Uhealth.

[23] Carbo A, Gupta M, Tamariz L, Palacio A, Levis S, Nemeth Z, Dang S (2018). Mobile Technologies for Managing Heart Failure: A Systematic Review and Meta-analysis. Telemed J E Health.

[24] Conway A, Inglis SC, Clark RA (2014). Effective technologies for noninvasive remote monitoring in heart failure. Telemed J E Health.

[25] Van Spall HG, Rahman T, Mytton O, Ramasundarahettige C, Ibrahim Q, Kabali C, Coppens M, Brian Haynes R, Connolly S (2017). Comparative effectiveness of transitional care services in patients discharged from the hospital with heart failure: a systematic review and network meta-analysis. Eur J Heart Fail.

[26] Higgins JT, Chandler J, Cumpston M, Li T, Page M (2020). Welch VA Cochrane Handbook for Systematic Reviews of Interventions version 6.0 (updated July 2019).

[27] Schwamm LH, Chumbler N, Brown E, Fonarow GC, Berube D, Nystrom K, Suter R, Zavala M, Polsky D, Radhakrishnan K, Lacktman N, Horton K, Malcarney MB, Halamka J, Tiner AC, American Heart Association Advocacy Coordinating Committee (2017). Recommendations for the Implementation of Telehealth in Cardiovascular and Stroke Care: A Policy Statement From the American Heart Association. Circulation.

[28] Meystre S (2005). The current state of telemonitoring: a comment on the literature. Telemed J E Health.

[29] Zhang Z, Xu X, Ni H (2013). Small studies may overestimate the effect sizes in critical care meta-analyses: a meta-epidemiological study. Crit Care.

[30] IntHout J, Ioannidis JP, Borm GF, Goeman JJ (2015). Small studies are more heterogeneous than large ones: a meta-meta-analysis. J Clin Epidemiol.

[31] Dechartres A, Trinquart L, Boutron I, Ravaud P (2013). Influence of trial sample size on treatment effect estimates: meta-epidemiological study. BMJ.

[32] Morgan JM, Kitt S, Gill J, McComb JM, Ng GA, Raftery J, Roderick P, Seed A, Williams SG, Witte KK, Wright DJ, Harris S, Cowie MR (2017). Remote management of heart failure using implantable electronic devices. Eur Heart J.

[33] Klersy C, Boriani G, De Silvestri A, Mairesse GH, Braunschweig F, Scotti V, Balduini A, Cowie MR, Leyva F, Health Economics Committee of the European Heart Rhythm Association (2016). Effect of telemonitoring of cardiac implantable electronic devices on healthcare utilization: a meta-analysis of randomized controlled trials in patients with heart failure. Eur J Heart Fail.

[34] DerSimonian R, Laird N (1986). Meta-analysis in clinical trials. Control Clin Trial.

[35] Jackson D, White IR, Thompson SG (2010). Extending DerSimonian and Laird's methodology to perform multivariate random effects meta-analyses. Stat Med.

[36] Viechtbauer W (2010). Conducting Meta-Analyses in R with the metafor Package. J Stat Soft.

[37] Higgins JPT, Thompson SG, Deeks JJ, Altman DG (2003). Measuring inconsistency in meta-analyses. BMJ.

[38] Dickersin K, Berlin Ja (1992). Meta-analysis: state-of-the-science. Epidemiol Rev.

[39] Sterne JAC, Sutton AJ, Ioannidis JPA, Terrin N, Jones DR, Lau J, Carpenter J, Rücker G, Harbord RM, Schmid CH, Tetzlaff J, Deeks JJ, Peters J, Macaskill P, Schwarzer G, Duval S, Altman DG, Moher D, Higgins JPT (2011). Recommendations for examining and interpreting funnel plot asymmetry in meta-analyses of randomised controlled trials. BMJ.

[40] (2015). RStudio: Integrated Development for R.

[41] Yun JE, Park J, Park H, Lee H, Park D (2018). Comparative Effectiveness of Telemonitoring Versus Usual Care for Heart Failure: A Systematic Review and Meta-analysis. J Card Fail.

[42] Scherr D, Kastner P, Kollmann A, Hallas A, Auer J, Krappinger H, Schuchlenz H, Stark G, Grander W, Jakl G, Schreier G, Fruhwald FM, MOBITEL Investigators (2009). Effect of home-based telemonitoring using mobile phone technology on the outcome of heart failure patients after an episode of acute decompensation: randomized controlled trial. J Med Internet Res.

[43] Bekelman DB, Plomondon ME, Carey EP, Sullivan MD, Nelson KM, Hattler B, McBryde CF, Lehmann KG, Gianola K, Heidenreich PA, Rumsfeld JS (2015). Primary Results of the Patient-Centered Disease Management (PCDM) for Heart Failure Study: A Randomized Clinical Trial. JAMA Intern Med.

[44] Benatar D, Bondmass M, Ghitelman J, Avitall B (2003). Outcomes of chronic heart failure. Arch Intern Med.

[45] Blum K, Gottlieb SS (2014). The effect of a randomized trial of home telemonitoring on medical costs, 30-day readmissions, mortality, and health-related quality of life in a cohort of community-dwelling heart failure patients. J Card Fail.

[46] Capomolla S, Pinna G, Larovere M, Maestri R, Ceresa M, Ferrari M, Febo O, Caporotondi A, Guazzotti G, Lenta F (2004). Heart failure case disease management program: a pilot study of home telemonitoring versus usual care. Eur Heart J Supp.

[47] Cleland J, Louis A, Rigby A, Janssens U, Balk A (2005). Noninvasive Home Telemonitoring for Patients With Heart Failure at High Risk of Recurrent Admission and Death: The Trans-European Network–Home-Care Management System (TEN-HMS) Study. ACC Curr J Rev.

[48] Comín-Colet J, Enjuanes C, Verdú-Rotellar JM, Linas A, Ruiz-Rodriguez P, González-Robledo G, Farré N, Moliner-Borja P, Ruiz-Bustillo S, Bruguera J (2015). Impact on clinical events and healthcare costs of adding telemedicine to multidisciplinary disease management programmes for heart failure: Results of a randomized controlled trial. J Telemed Telecare.

[49] Dar O, Riley J, Chapman C, Dubrey SW, Morris S, Rosen SD, Roughton M, Cowie MR (2009). A randomized trial of home telemonitoring in a typical elderly heart failure population in North West London: results of the Home-HF study. Eur J Heart Fail.

[50] Dendale P, De Keulenaer G, Troisfontaines P, Weytjens C, Mullens W, Elegeert I, Ector B, Houbrechts M, Willekens K, Hansen D (2012). Effect of a telemonitoring-facilitated collaboration between general practitioner and heart failure clinic on mortality and rehospitalization rates in severe heart failure: the TEMA-HF 1 (TElemonitoring in the MAnagement of Heart Failure) study. Eur J Heart Fail.

[51] Giordano A, Scalvini S, Zanelli E, Corrà U, Longobardi GL, Ricci V, Baiardi P, Glisenti F (2009). Multicenter randomised trial on home-based telemanagement to prevent hospital readmission of patients with chronic heart failure. Int J Cardiol.

[52] Goldberg LR, Piette JD, Walsh MN, Frank TA, Jaski BE, Smith AL, Rodriguez R, Mancini DM, Hopton LA, Orav E, Loh E (2003). Randomized trial of a daily electronic home monitoring system in patients with advanced heart failure: the Weight Monitoring in Heart Failure (WHARF) trial. Am Heart J.

[53] Hägglund E, Lyngå P, Frie F, Ullman B, Persson H, Melin M, Hagerman I (2015). Patient-centred home-based management of heart failure. Findings from a randomised clinical trial evaluating a tablet computer for self-care, quality of life and effects on knowledge. Scand Cardiovasc J.

[54] Kalter-Leibovici O, Freimark D, Freedman LS, Kaufman G, Ziv A, Murad H, Benderly M, Silverman BG, Friedman N, Cukierman-Yaffe T, Asher E, Grupper A, Goldman D, Amitai M, Matetzky S, Shani M, Silber H, Israel Heart Failure Disease Management Study (IHF-DMS) investigators (2017). Disease management in the treatment of patients with chronic heart failure who have universal access to health care: a randomized controlled trial. BMC Med.

[55] Koehler F, Koehler K, Deckwart O, Prescher S, Wegscheider K, Kirwan B, Winkler S, Vettorazzi E, Bruch L, Oeff M, Zugck C, Doerr G, Naegele H, Störk S, Butter C, Sechtem U, Angermann C, Gola G, Prondzinsky R, Edelmann F, Spethmann S, Schellong SM, Schulze PC, Bauersachs J, Wellge B, Schoebel C, Tajsic M, Dreger H, Anker SD, Stangl K (2018). Efficacy of telemedical interventional management in patients with heart failure (TIM-HF2): a randomised, controlled, parallel-group, unmasked trial. Lancet.

[56] Kotooka N, Kitakaze M, Nagashima K, Asaka M, Kinugasa Y, Nochioka K, Mizuno A, Nagatomo D, Mine D, Yamada Y, Kuratomi A, Okada N, Fujimatsu D, Kuwahata S, Toyoda S, Hirotani S, Komori T, Eguchi K, Kario K, Inomata T, Sugi K, Yamamoto K, Tsutsui H, Masuyama T, Shimokawa H, Momomura S, Seino Y, Sato Y, Inoue T, Node K, HOMES-HF study investigators (2018). The first multicenter, randomized, controlled trial of home telemonitoring for Japanese patients with heart failure: home telemonitoring study for patients with heart failure (HOMES-HF). Heart Vessels.

[57] Lyngå P, Persson H, Hägg-Martinell A, Hägglund E, Hagerman I, Langius-Eklöf A, Rosenqvist M (2012). Weight monitoring in patients with severe heart failure (WISH). A randomized controlled trial. Eur J Heart Fail.

[58] Melin M, Hägglund Ewa, Ullman B, Persson H, Hagerman I (2018). Effects of a Tablet Computer on Self-care, Quality of Life, and Knowledge: A Randomized Clinical Trial. J Cardiovasc Nurs.

[59] Mortara A, Pinna GD, Johnson P, Maestri R, Capomolla S, La Rovere MT, Ponikowski P, Tavazzi L, Sleight P, HHH Investigators (2009). Home telemonitoring in heart failure patients: the HHH study (Home or Hospital in Heart Failure). Eur J Heart Fail.

[60] Olivari Z, Giacomelli S, Gubian L, Mancin S, Visentin E, Di Francesco V, Iliceto S, Penzo M, Zanocco A, Marcon C, Anselmi M, Marchese D, Stafylas P (2018). The effectiveness of remote monitoring of elderly patients after hospitalisation for heart failure: The renewing health European project. Int J Cardiol.

[61] Seto E, Leonard KJ, Cafazzo JA, Barnsley J, Masino C, Ross HJ (2012). Mobile phone-based telemonitoring for heart failure management: a randomized controlled trial. J Med Internet Res.

[62] Soran OZ, Piña IL, Lamas GA, Kelsey SF, Selzer F, Pilotte J, Lave JR, Feldman AM (2008). A randomized clinical trial of the clinical effects of enhanced heart failure monitoring using a computer-based telephonic monitoring system in older minorities and women. J Card Fail.

[63] Villani A, Malfatto G, Compare A, Della Rosa F, Bellardita L, Branzi G, Molinari E, Parati G (2014). Clinical and psychological telemonitoring and telecare of high risk heart failure patients. J Telemed Telecare.

[64] Wade MJ, Desai AS, Spettell CM, Snyder AD, McGowan-Stackewicz V, Kummer PJ, Maccoy MC, Krakauer RS (2011). Telemonitoring with case management for seniors with heart failure. Am J Manag Care.

[65] Wagenaar KP, Broekhuizen BD, Jaarsma T, Kok I, Mosterd A, Willems FF, Linssen GC, Agema WR, Anneveldt S, Lucas CM, Mannaerts HF, Wajon EM, Dickstein K, Cramer MJ, Landman MA, Hoes AW, Rutten FH (2019). Effectiveness of the European Society of Cardiology/Heart Failure Association website 'heartfailurematters.org' and an e-health adjusted care pathway in patients with stable heart failure: results of the 'e-Vita HF' randomized controlled trial. Eur J Heart Fail.

[66] Sacks CA, Jarcho JA, Curfman GD (2014). Paradigm shifts in heart-failure therapy--a timeline. N Engl J Med.

[67] Ruppar TM, Cooper PS, Mehr DR, Delgado JM, Dunbar-Jacob JM (2016). Medication Adherence Interventions Improve Heart Failure Mortality and Readmission Rates: Systematic Review and Meta-Analysis of Controlled Trials. J Am Heart Assoc.

[68] Zhang Y, Wu S, Fendrick AM, Baicker K (2013). Variation in medication adherence in heart failure. JAMA Intern Med.

[69] Cajita MI, Hodgson NA, Budhathoki C, Han H (2017). Intention to Use mHealth in Older Adults With Heart Failure. J Cardiovasc Nurs.

[70] Anderson K, Burford O, Emmerton L (2016). Mobile Health Apps to Facilitate Self-Care: A Qualitative Study of User Experiences. PLoS One.

[71] Klasnja P, Pratt W (2012). Healthcare in the pocket: mapping the space of mobile-phone health interventions. J Biomed Inform.

[72] Bashi N, Karunanithi M, Fatehi F, Ding H, Walters D (2017). Remote Monitoring of Patients With Heart Failure: An Overview of Systematic Reviews. J Med Internet Res.

[73] Varnfield M, Karunanithi M, Lee C, Honeyman E, Arnold D, Ding H, Smith C, Walters DL (2014). Smartphone-based home care model improved use of cardiac rehabilitation in postmyocardial infarction patients: results from a randomised controlled trial. Heart.

[74] Athilingam P, Jenkins B, Johansson M, Labrador M (2017). A Mobile Health Intervention to Improve Self-Care in Patients With Heart Failure: Pilot Randomized Control Trial. JMIR Cardio.

[75] Piette JD, Striplin D, Marinec N, Chen J, Trivedi RB, Aron DC, Fisher L, Aikens JE (2015). A Mobile Health Intervention Supporting Heart Failure Patients and Their Informal Caregivers: A Randomized Comparative Effectiveness Trial. J Med Internet Res.

[76] Burke LE, Ma J, Azar KM, Bennett GG, Peterson ED, Zheng Y, Riley W, Stephens J, Shah SH, Suffoletto B, Turan TN, Spring B, Steinberger J, Quinn CC, American Heart Association Publications Committee of the Council on EpidemiologyPrevention‚ Behavior Change Committee of the Council on Cardiometabolic Health‚ Council on CardiovascularStroke Nursing‚ Council on Functional GenomicsTranslational Biology‚ Council on Quality of CareOutcomes Research‚Stroke Council (2015). Current Science on Consumer Use of Mobile Health for Cardiovascular Disease Prevention: A Scientific Statement From the American Heart Association. Circulation.

[77] Ali EE, Chew L, Yap KY (2016). Evolution and current status of mhealth research: a systematic review. BMJ Innov.

[78] Masterson Creber RM, Maurer MS, Reading M, Hiraldo G, Hickey KT, Iribarren S (2016). Review and Analysis of Existing Mobile Phone Apps to Support Heart Failure Symptom Monitoring and Self-Care Management Using the Mobile Application Rating Scale (MARS). JMIR Mhealth Uhealth.

