# Supplementary figures and images for: Effects of Different Telemonitoring Strategies on Chronic Heart Failure Care: Systematic Review and Subgroup Meta-Analysis
Source: J Med Internet Res. 2020 Nov 13;22(11):e20032. doi: 10.2196/20032 (PMC7695537; doi:10.2196/20032)

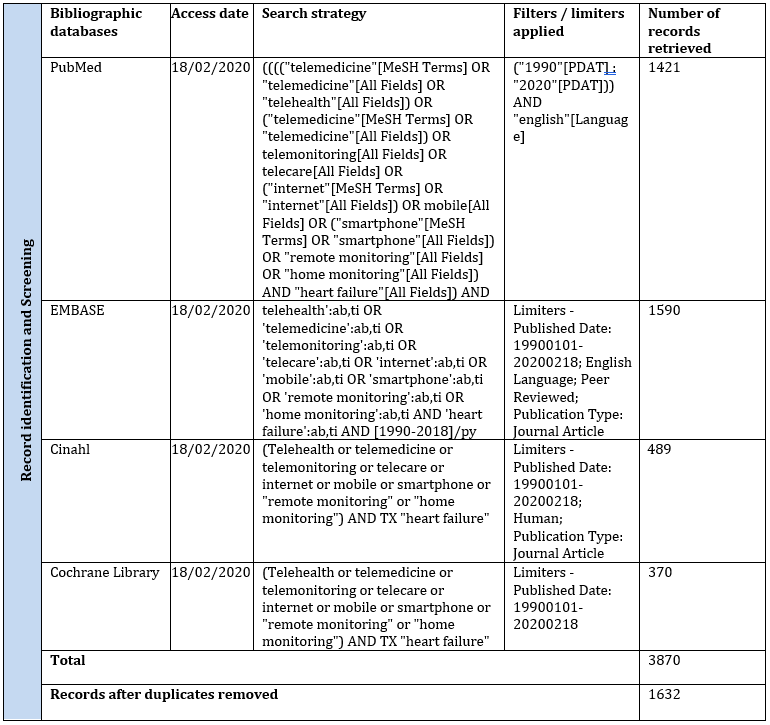

Supplement: Multimedia Appendix 1 [file jmir_v22i11e20032_app1.png]

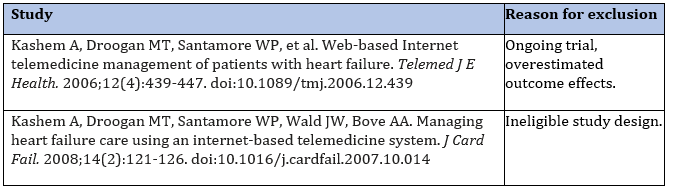

Supplement: Multimedia Appendix 2 [file jmir_v22i11e20032_app2.png]

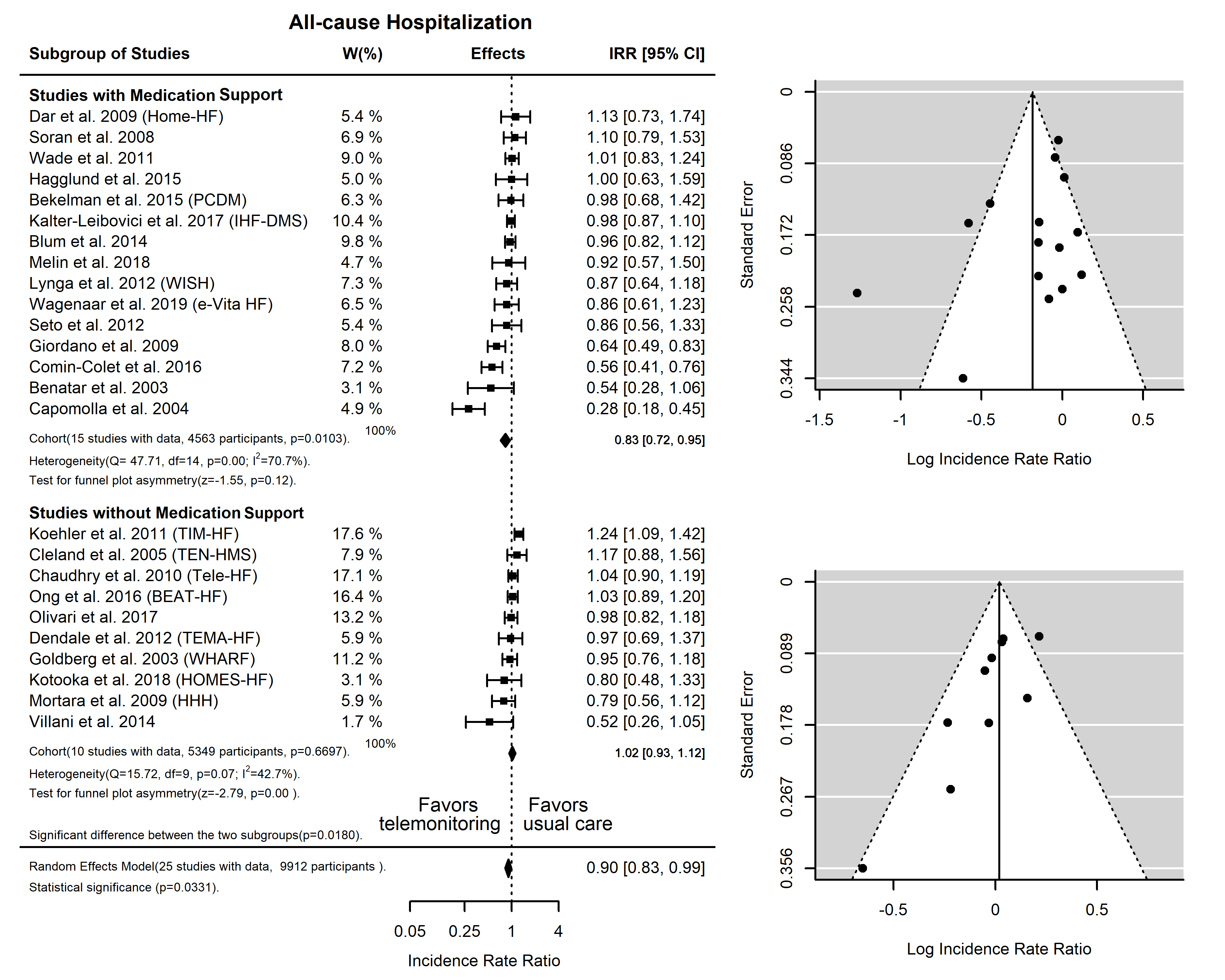

Supplement: Multimedia Appendix 3 [file jmir_v22i11e20032_app3.png]

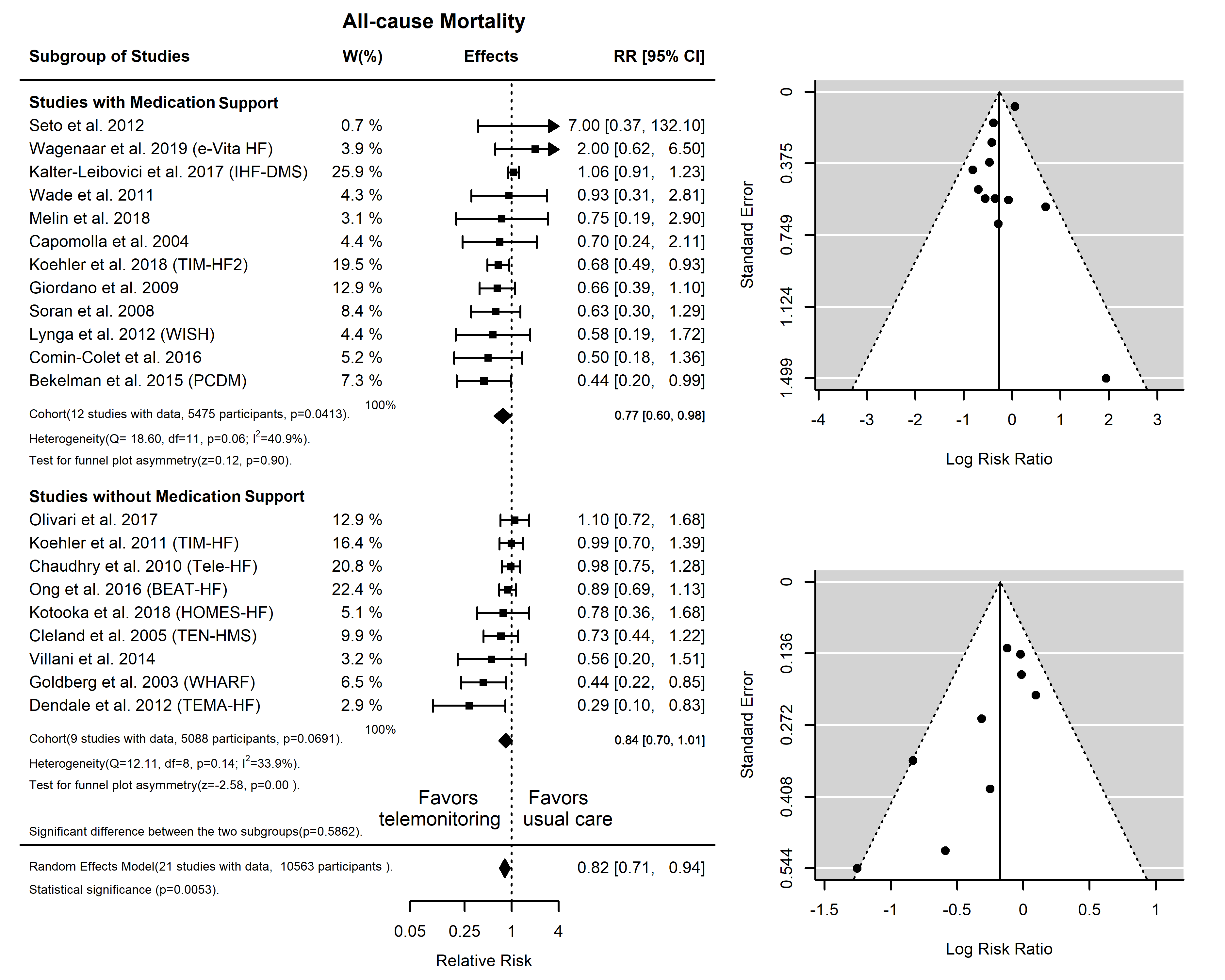

Supplement: Multimedia Appendix 4 [file jmir_v22i11e20032_app4.png]

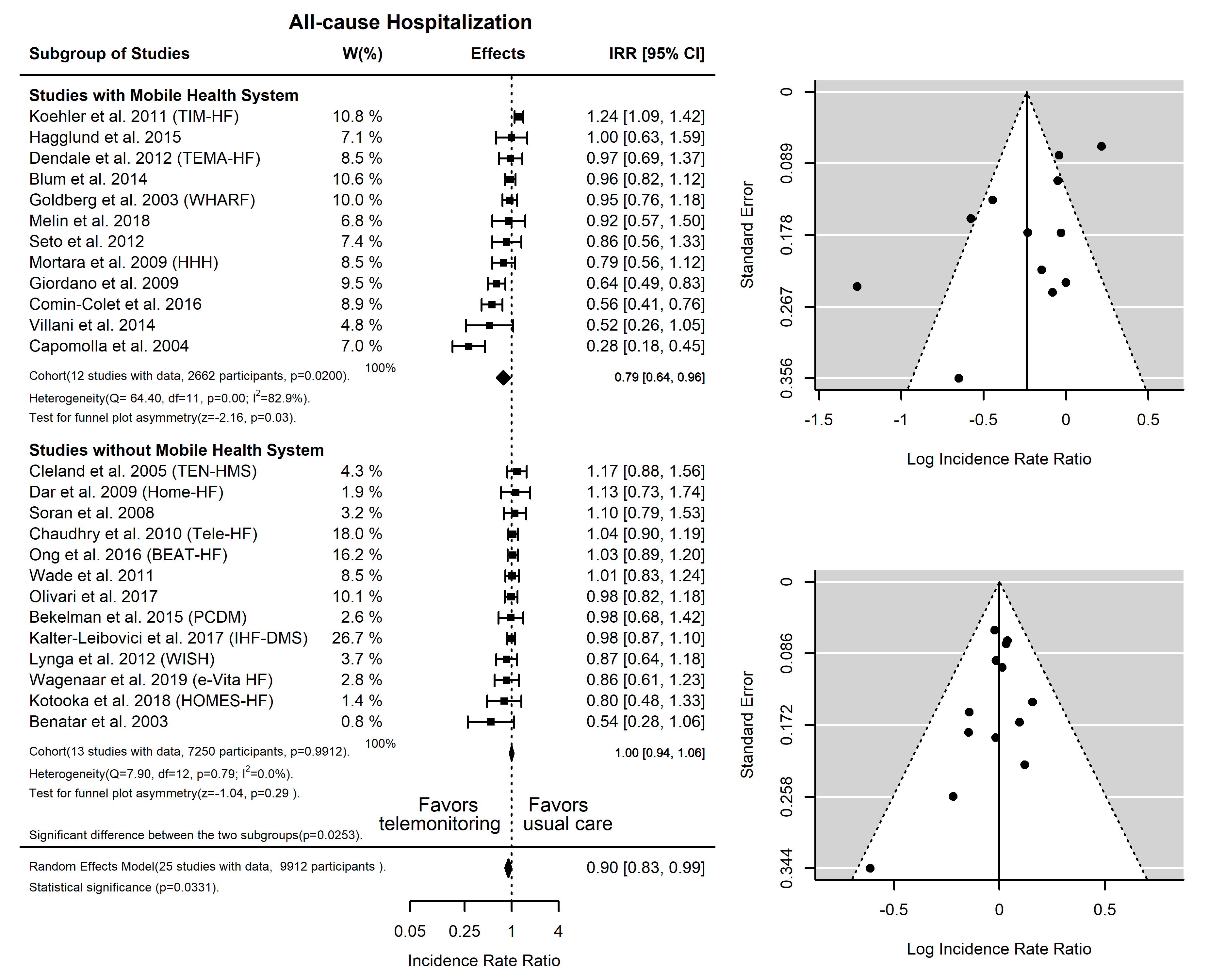

Supplement: Multimedia Appendix 5 [file jmir_v22i11e20032_app5.png]

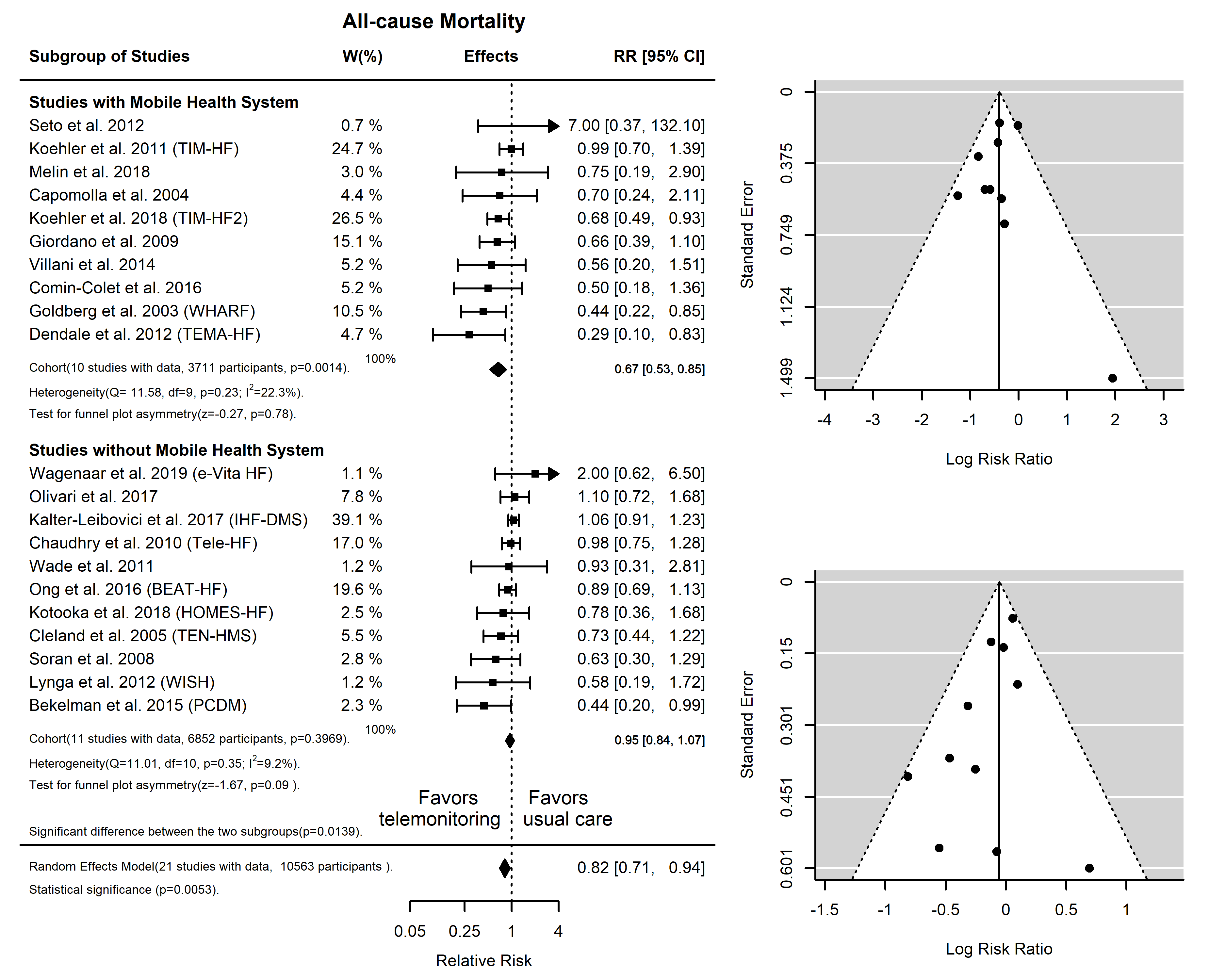

Supplement: Multimedia Appendix 6 [file jmir_v22i11e20032_app6.png]
